# Comparing the effectiveness of group-based Cognitive-Behavioral Couple Therapy (CBCT) and Schema-Based Couple Therapy (SBCT) *workshops* in reducing desire for divorce and marital boredom

**DOI:** 10.1186/s13104-025-07339-4

**Published:** 2025-07-05

**Authors:** Azam Ardeh, Elham Goodarzi, Zahra Madadi, Changiz Rostami, Ali Ghiaci, Jamile Shahverdi

**Affiliations:** 1 Department of Clinical Psychology, School of Public Health, Shahid Sadoughi University of Medical Sciences, Yazd, Iran; 2 Social Determinants of Health Research Center, Lorestan University of Medical Sciences, Khoramabad, Iran; 3 Social Determinants of Health Research Center, Iran University of Medical Sciences, Tehran, Iran; 4 Department of Management Sciences and Health Economics, School of Public Health, Tehran University of Medical Sciences, Tehran, Iran; 5 School of Medicine, Iran University of Medical Sciences, Tehran, Iran

**Keywords:** Cognitive-behavioral couple therapy, Schema-based couple therapy, Desire for divorce, Marital boredom

## Abstract

**Background:**

Marital relationships, like any other interpersonal bond, encompass both positive and negative dimensions. Central to the success of marriage is the level of satisfaction and commitment experienced by the partners. This study aims to compare the efficacy of cognitive-behavioral couple therapy and schema-based couple therapy in reducing the desire for divorce and marital boredom.

**Methods:**

This experimental study employed a pre-test-post-test design with a control group. The research sample comprised couples experiencing marital issues who sought counseling at various counseling centers in 2023. Sixty couples were randomly assigned to three groups: two intervention groups and one control group. Data were collected using questionnaires assessing desire for divorce (14 questions) and marital boredom (21 questions). The intervention consisted of 10 weekly sessions lasting 90 min each, conducted in group format for the two intervention groups. Questionnaire data were collected prior to the intervention, one week post-intervention, and one month post-intervention. Data analysis involved the use of analysis of variance and repeated measures tests to assess changes over time.

**Results:**

The results indicate a significant decrease in the average boredom scores in both the cognitive-behavioral and schema therapy groups following the intervention (*P* < 0.0001). Conversely, no significant change was observed in the control group before and after the intervention (*P* > 0.05). Repeated measures analysis revealed a significant difference in the mean boredom scores before the intervention, post-intervention, and one month later in both the cognitive-behavioral and schema therapy groups (*P* < 0.0001). In contrast, the control group exhibited stable mean boredom scores across the three assessment points (*P* > 0.05). Additionally, there was a significant decrease in the average scores of the desire for divorce in both the cognitive-behavioral and schema therapy groups post-intervention (*P* < 0.0001), while no significant difference was observed in the control group before and after the intervention (*P* > 0.05). Similar to the boredom scores, repeated measures analysis demonstrated a significant difference in the mean divorce scores across the three assessment points in the intervention groups, whereas scores remained stable in the control group (*P* > 0.05).

**Conclusion:**

The findings of this study suggest that both therapy methods are effective in reducing the desire for divorce and marital boredom. These treatment approaches can be valuable tools in family therapy settings aimed at enhancing the commitment of couples.

## Introduction

The family, a dynamic institution, continuously adapts to societal shifts while retaining an inherent stability to navigate through changes [1]. The resilience and cohesion of a family unit depend on the robustness of marital bonds; any lack of satisfaction or relational success between spouses can negatively impact their mental well-being, thus endangering the longevity and solidity of the family structure [2].

In recent times, divorce has become a prevalent global concern, with the breakdown of family foundations at its core. Sociologists consider divorce a substantial social problem, interpreting rising divorce rates as indicative of moral decline, decreased family harmony, interpersonal conflicts, and disruptions in societal norms [3]. Throughout the 20th century, driven by population growth and cultural changes, especially in industrialized nations, divorce rates have consistently increased [4].

Psychologists explore individual-level factors contributing to divorce, while sociologists examine community-level dynamics. Psychological frameworks emphasize intrapersonal behaviors, personality traits, childhood experiences, and cognitive schemas [5].

Analyzing data from the national civil registry reveals a steady increase in divorce rates in Iran since 2000. The figures have risen from 60 thousand divorces in 2000 to 183,193 in 2020. Reports from the Center for Asian Population Studies and Research support this upward trend in divorce rates, particularly pronounced in urban areas compared to rural regions. In 2019, urban areas recorded 170,688 divorces, contrasting with 12,505 in rural areas [6].

Marital boredom arises as a notable contributor to couples’ divorce. It is defined by a sense of physical, mental, and emotional weariness, occurring when couples come to the realization that their relationship, despite sincere efforts, no longer offers the meaning and fulfillment they seek in their lives [7].

Couples’ therapy has seen a proliferation of treatment modalities tailored to address communication conflicts, encompassing cognitive-behavioral Couples’ therapy, systemic therapy, acceptance and commitment therapy, emotion-focused Couples’ therapy, solution-focused therapy, schema couple therapy, and more. Among these, cognitive-behavioral couple therapy stands out as a potent intervention, integrating behavior modification techniques like communication enhancement skills with cognitive restructuring. Over the past decade, cognitive-behavioral couple therapy has emerged as a highly effective strategy in addressing communication issues, with a focus on enhancing couples’ problem-solving abilities through cognitive restructuring and improved communication [8, 9]. A modern counseling approach gaining momentum is Yang’s schema therapy, esteemed for its effectiveness in uncovering marital obstacles. Grounded in the cognitive paradigm, this theory explores the fundamental incompatible schemas that underlie familial and marital strife [10].

Schema-based therapy aims to identify and address marital challenges by helping couples recognize, challenge, and modify maladaptive schemas through targeted techniques. These schemas, rooted in early life, mold individuals’ cognitions, shaping how they perceive and interpret reality. Often, individuals unknowingly align their worldview with these schemas, which originate from adverse childhood experiences and endure into adulthood [8, 9].

Recent research underscores the efficacy of cognitive-behavioral therapy and schema-based couple therapy in enhancing outcomes within couple therapy. Consequently, this study seeks to compare the effectiveness of cognitive-behavioral couple (CBCT) and schema-based couple therapy in alleviating the inclination towards divorce and reducing marital boredom.

## Methods

Because this study is a non-pharmacological interventional study, it was designed and conducted according to the CONSORT guidelines for non-pharmacological randomized trials [11]. The study employed a experimental pre-test-post-test design involving three groups (two intervention groups and one control group).

The statistical population of this study was all couples who visited counseling centers in Boroujerd city with marital problems in 2023. The research sample was selected randomly from couples who voluntarily registered after being invited to couple therapy. The sample size was calculated with the formula of 16 couples for each group [12]. However, due to sample size reduction, 20 couples were considered for each group. Finally, 60 couples (group of 20 couples) were randomly selected and placed into three groups using simple randomization.

Inclusion criteria encompassed couples contemplating divorce, aged between 20 and 50 years, possessing minimal education, and providing informed consent. Exclusion criteria involved chronic physical illnesses, severe mental disorders such as schizophrenia, depression, bipolar disorder, substance abuse, missing more than two intervention sessions, failure to complete therapy homework diligently, expressing reluctance to engage, and incomplete questionnaire responses.

After the subjects were divided into three groups, the first group was taught Cognitive-Behavioral Couple Therapy(CBCT) and the second group was taught schema-based couple therapy (both groups received intervention in the form of training classes), and no training was provided to the third group. At the end of each session, homework was given to the two intervention groups. The intervention was implemented through educational classes. And at the end of each session, a review of the contents of the previous session was conducted. A summary of the intervention sessions is reported in Tables 1 and 2 [13, 14].

The statistical population of this study was all couples who visited counseling centers in Boroujerd city with marital problems in 2023. The research sample was selected randomly from couples who voluntarily registered after being invited to couple therapy. The sample size was calculated with the formula of 16 couples for each group [12]. However, due to sample size reduction, 20 couples were considered for each group. Finally, 60 couples (group of 20 couples) were randomly selected and placed into three groups using simple randomization.

Inclusion criteria encompassed couples contemplating divorce, aged between 20 and 50 years, possessing minimal education, and providing informed consent. Exclusion criteria involved chronic physical illnesses, severe mental disorders such as schizophrenia, depression, bipolar disorder, substance abuse, missing more than two intervention sessions, failure to complete therapy homework diligently, expressing reluctance to engage, and incomplete questionnaire responses.

**Fig. 1:**
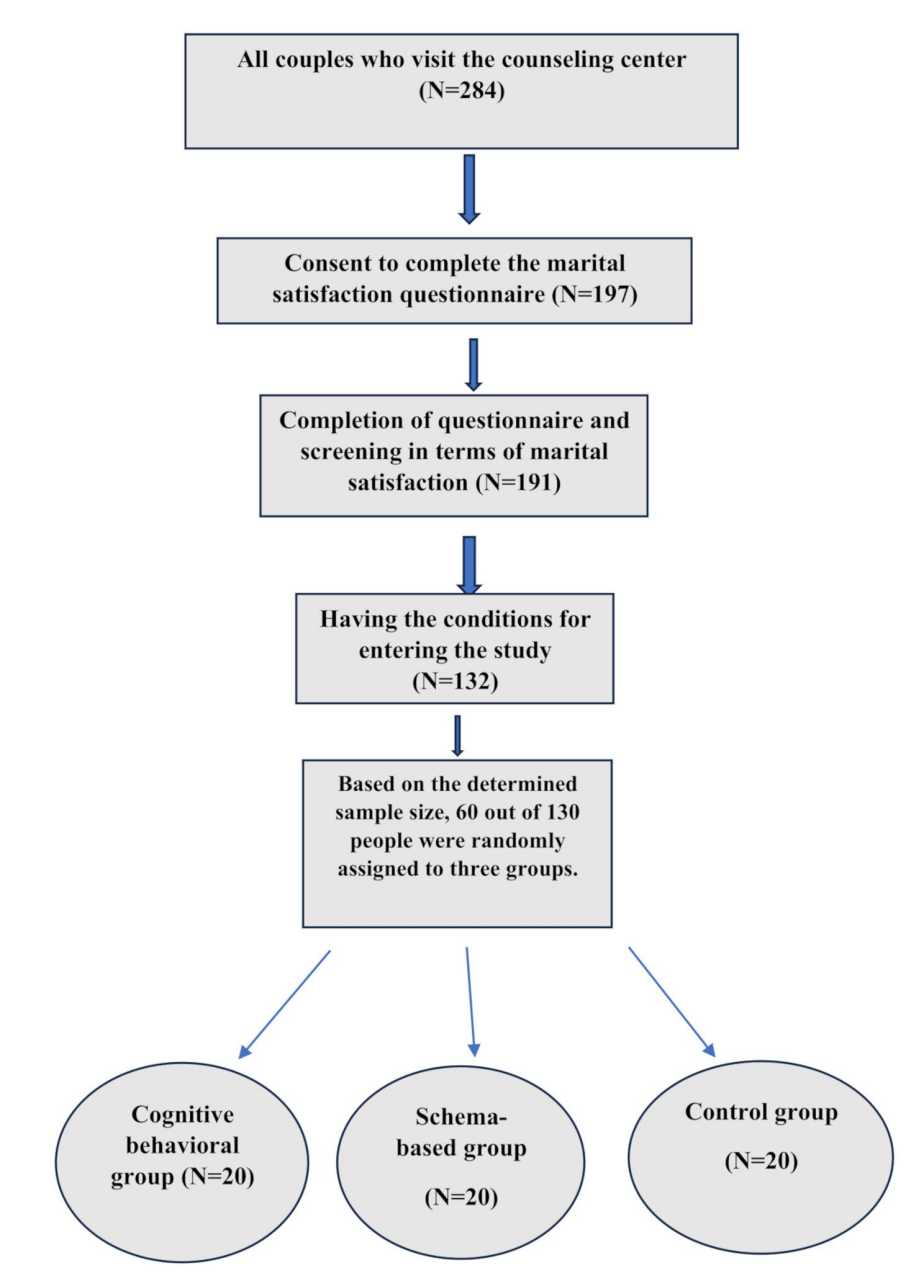
Diagram of participant flowchart

After the subjects were divided into three groups, the first group was taught Cognitive-Behavioral Couple Therapy(CBCT) and the second group was taught schema-based couple therapy (both groups received intervention in the form of training classes), and no training was provided to the third group. At the end of each session, homework was given to the two intervention groups. The intervention was implemented through educational classes. And at the end of each session, a review of the contents of the previous session was conducted. The method of enrolling participants in the study is explained in Fig. 1. A summary of the intervention sessions is reported in Tables 1 and 2 [13, 14].

**Table 1:** Structure of Cognitive-Behavioral couple therapy (CBCT) sessions’ content

**First session**	Couples engage in introductions, establish group goals and guidelines, familiarizing themselves with cognitive-behavioral therapy principles, define marital quality, and writing expectations from their spouse and married life.
**Second session**	Elucidating the core tenets of the cognitive-behavioral therapy model, identifying both positive and negative traits within themselves, their partner, and their marriage.
**Third session**	Pinpointing the couple’s beliefs and unrealistic expectations by employing cognitive skills training.
**Fourth session**	Understanding the role of cognitive factors and cognitive errors, working towards rectifying any cognitive distortions present.
**Fifth session**	exploring the concept of schemas and schema styles, examining the impact of schemas on marital issues and processing associated emotions.
**Sixth session**	Learning communication fundamentals, emphasizing the importance of effective verbal and non-verbal interactions, and fostering the open exchange of emotions with one’s spouse.
**Seventh session**	Learning about reinforcement and punishment patterns within the relationship, emphasizing the importance of positive reinforcement and enhancing behavioral exchanges.
**Eighth session**	Learning the six preventive aspects of the cognitive-behavioral model
**Ninth session**	Learning problem-solving skills such as avoidance, persuasion, compromise, indecisiveness, and collaboration.
**Tenth session**	summarizing key learnings, gathering feedback, drawing conclusions, reinforcing treatment effects, and conducting post-test assessments.

In Schema-based couple therapy sessions, drawing from Yang’s Schema Therapy book, participants engaged in a series of ten 90-minute sessions designed to address relational challenges. The specifics of these sessions are outlined in Table 2 [15].

**Table 2:** Structure of Schema-Based couple therapy (SBCT) sessions’ content

Session	Purpose	Content
**First**	Familiarization, establishing communication and empathy	Introducing the participants and the therapist to each other, stating the rules and regulations of the group, getting to know schema therapy and its process.
**Second**	Knowing the central needs and evolutionary roots of schemas	Formulating problems in the form of schema therapy, getting to know the six categories of primary central needs and the evolutionary roots of schemas and how they are formed.
**Third**	Knowing the domains and schemas	Knowing the five domains and eighteen primary incompatible schemas and their role in marital problems.
**Fourth**	Knowing the characteristics of different types of schemas	Knowing the characteristics of five domains and eighteen primary incompatible schemas, their role in marital boredom and the desire for divorce.
**Fifth**	Knowing the conditional and unconditional schemas	Identifying the conditional and unconditional schemas of couples applying for divorce and discussing how they are formed and their effects and consequences.
**Sixth**	Knowing how schemas continue	Teaching how schemas work, how to maintain and continue them, and examine their effects and consequences.
**Seventh**	Knowing ineffective coping styles	Knowing ineffective coping styles during marital conflicts and highlighting them in marital life
**Eighth**	Knowing the role of schemas in life	Examining the role of schemas in marital relationships and highlighting them in marital life
**Ninth**	Learning to adjust and reduce the impact of ineffective schemas	Learning to deal with ineffective schemas and reduce their negative effects through checking the validity of schemas, evaluating the advantages and disadvantages of coping styles and distinguishing between responses to schemas
**Tenth**	Learning to adjust and reduce the impact of ineffective schemas	Learning to deal with ineffective schemas and reduce their negative effects through writing letters, preparing training cards, imaginary conversations and playing roles in real life situations.

### Measurement tools

#### Enrich marital satisfaction questionnaire

In this study, the Enrich Marital Satisfaction Scale (1989), a 47-item questionnaire was utilized. The questionnaire employs a 5-point Likert scale, with response options ranging from is scored from 1 to 5 (Strongly Disagree to Strongly Agree (, resulting in a minimum total score of 47 and a maximum of 235. A higher score on the questionnaire indicates greater marital satisfaction among couples. Olson reported the reliability of the Enrich questionnaire using Cronbach’s alpha coefficient as 0.92 [16].

#### Marital boredom questionnaire

The Marital Boredom Questionnaire, developed by Pines (1996), comprises 21 questions designed to assess symptoms of burnout within marital relationships. The questionnaire encompasses three categories: emotional fatigue, mental fatigue, and physical fatigue. Scores on this scale range from 21 to 147, with higher scores indicative of greater levels of marital boredom.

Test-retest reliability coefficients for this questionnaire were reported as 0.89 over a one-month interval, 0.76 over a two-month interval, and 0.66 over a four-month interval. Additionally, Cronbach’s alpha coefficients ranged between 0.91 and 0.93 [17, 18].

#### Willingness to divorce questionnaire

The Willingness to Divorce Questionnaire, developed by Rusbult et al., comprises 14 questions aimed at assessing the propensity of couples toward divorce. The questionnaire evaluates cognitive, emotional, and behavioral categories of divorce inclination, in which the cognitive category includes the person’s perception on divorce and in the emotional category includes the positive and negative feelings and emotions of the person towards the divorce, and the behavioral category includes the level of a person’s behavior towards or against divorce. The reliability of this questionnaire, assessed using Cronbach’s alpha method, yielded coefficients of 0.91 for divorce inclination and 0.86 for divorce tolerance [18, 19].

Data analysis was conducted using Stata-17 software with a significance level set at 0.05. Analytical procedures involved verifying data normality using the Kolmogorov-Smirnov test. Statistical tests, including analysis of variance (ANOVA), were employed to assess differences between groups and repeated measurements over time (pre-intervention, post-intervention, and one month later) for each group.

## Results

The study’s findings revealed that there were no significant statistical differences in the demographic variables among the couples in the cognitive-behavioral group, the schema group, and the control group, indicating homogeneity across the three study groups (Table 3).

**Table 3:** Distribution of couples’ demographic variables in study groups

Variable		Frequency (percentage)			Chi-2	P-value
		Cognitive behavioral group (*n* = 20)	Schema-based group (*n* = 20)	Control group (*n* = 20)		
**Age**	20–30	9 (45)	8 (40)	8 (40)	0.1	0.7
	30–40	11 (55)	12 (60)	12 (60)		
**Occupation**	Employee	8 (40)	7 (35)	9 (45)	0.1	0.74
	Self-employed	12 (60)	13 (65)	11 (55)		
**Education**	Under diploma	8 (40)	6 (30)	7 (35)	0.3	0.58
	Diploma	5 (25)	7 (35)	6 (30)		
	Over diploma	7 (35)	7 (35)	7 (35)		
**Children**	No children	5 (25)	6 (30)	6 (30)	0.4	0.84
	1 child	4 (20)	6 (30)	5 (25)		
	> 1 children	11 (55)	8 (40)	9 (45)		
**Marriage duration**	2–5 years	9 (45)	8 (40)	9 (45)	0.13	0.93
	5–10 years	6 (30)	7 (35)	6 (30)		
	> 10 years	5 (25)	5 (25)	5 (25)		

The results of variance analysis indicated that the average marital satisfaction scores before the intervention were 57.63 in the cognitive-behavioral group, 58.02 in the schema group, and 56.9 in the control group, with no significant statistical difference observed (*P* = 0.762).

However, post-intervention, the average marital satisfaction scores decreased to 132.05 in the cognitive-behavioral group, 134.01 in the schema group, and 57.1 in the control group, revealing a significant difference between the groups (*p* < 0.0001).

Follow-up assessments indicated no significant difference in marital Satisfaction scores between the cognitive-behavioral and schema groups (*P* > 0.05), but both groups exhibited significant differences compared to the control group (*P* > 0.05), highlighting the efficacy of both interventions. Notably, marital Satisfaction scores significantly decreased post-intervention in both the cognitive-behavioral and schema groups (*P* < 0.0001), with a more substantial decrease observed in the cognitive-behavioral group, the control group showed no significant changes in marital Satisfaction scores pre- and post-intervention (*P* > 0.05).

Repeated measures analysis demonstrated significant differences in marital Satisfaction scores within the cognitive-behavioral and schema groups over time (*P* < 0.0001), with noticeable decreases observed post-intervention and one month later.

However, the differences between post-intervention and one-month follow-up were not statistically significant (*P* > 0.05) within these groups.

In contrast, the control group displayed consistent marital Satisfaction scores across the three stages of assessment (*P* > 0.05), But the average score before the intervention and one month after the intervention showed a statistically significant difference (*P* < 0.0001). In the control group, the changes in the average marital Satisfaction score in three stages were constant (*P* > 0.05). (Table 4; Fig. 2).

**Table 4:** Comparison of the average marital satisfaction scores in the groups and over time

Marriage boredom	Cognitive behavioral group	Schema-based group	Control group	F	*P*-value
**Before intervention**	57.63 ± 5.3	58.02 ± 4.7	56.9 ± 5.2	0.88	0.762
**Post-intervention**	132.05 ± 7.1	134.01 ± 6.8	57.1 ± 4.9	71.2	*P* < 0.0001
**One month after intervention**	131.76 ± 6.9	133.25 ± 6.6	56.8 ± 5.1	69.8	*P* < 0.0001
	*P* < 0.0001	*P* < 0.0001	*P* > 0.05		

The results of variance analysis indicated that the average boredom scores before the intervention were 2.83 in the cognitive-behavioral group, 2.81 in the schema group, and 2.88 in the control group, with no significant statistical difference observed (*P* = 0.386). However, post-intervention, the average boredom scores decreased to 1.45 in the cognitive-behavioral group, 1.5 in the schema group, and 2.84 in the control group, revealing a significant difference between the groups (*p* < 0.0001). Follow-up assessments indicated no significant difference in boredom scores between the cognitive-behavioral and schema groups (*P* > 0.05), but both groups exhibited significant differences compared to the control group (*P* > 0.05), highlighting the efficacy of both interventions. Notably, boredom scores significantly decreased post-intervention in both the cognitive-behavioral and schema groups (*P* < 0.0001), with a more substantial decrease observed in the cognitive-behavioral group, the control group showed no significant changes in boredom scores pre- and post-intervention (*P* > 0.05).

Repeated measures analysis demonstrated significant differences in boredom scores within the cognitive-behavioral and schema groups over time (*P* < 0.0001), with noticeable decreases observed post-intervention and one month later. However, the differences between post-intervention and one-month follow-up were not statistically significant (*P* > 0.05) within these groups. In contrast, the control group displayed consistent boredom scores across the three stages of assessment (*P* > 0.05), But the average score before the intervention and one month after the intervention showed a statistically significant difference (*P* < 0.0001). In the control group, the changes in the average score of boredom in three stages were constant (*P* > 0.05). (Table 5; Fig. 2).

**Table 5:** Comparison of the average boredom score in the groups and over time

Marriage boredom	Cognitive behavioral group	Schema-based group	Control group	F	*P*-value
**Before intervention**	2.83 ± 0.14	2.81 ± 0.11	2.88 ± 0.21	0.97	0.386
**Post-intervention**	1.45 ± 0.15	1.5 ± 0.15	2.84 ± 0.23	36.6	*P* < 0.0001
**One month after intervention**	1.44 ± 0.13	1.48 ± 0.15	2.8 ± 0.24	35.2	*P* < 0.0001
	*P* < 0.0001	*P* < 0.0001	*P* > 0.05		

The results of variance analysis indicated that the average desire for divorce score before the intervention was 59.85 in the cognitive-behavioral group, 63.85 in the schema group, and 57.6 in the control group, with no significant statistical difference noted among the three groups (*P* > 0.05). However, post-intervention, the average desire for divorce scores decreased to 36.9 in the cognitive-behavioral group, 36.3 in the schema group, and 58.1 in the control group, revealing a significant difference between the groups (*P* < 0.0001).

Follow-up assessments indicated no statistically significant difference in the average desire for divorce scores between the cognitive-behavioral and schema groups (*P* > 0.05). Nevertheless, both groups exhibited significant differences compared to the control group (*P* < 0.0001). Both the cognitive-behavioral and schema groups demonstrated a significant reduction in the average desire for divorce scores post-intervention, and this declination in the schema group was more than the cognitive-behavioral group, while in the control group before and after the intervention, no difference was observed in the average score of desire for divorce (*P* > 0.05).

The repeated measurements analysis highlighted significant differences in the average desire for divorce scores within the cognitive-behavioral and schema groups across the pre-intervention, post-intervention, and one-month follow-up periods. However, while the average score of the desire for divorce after the intervention and one month later did not exhibit a statistically significant difference, the disparity between the average score before the intervention and one month later indicated a notable variance (*P* < 0.0001). In the schema group, similarly, there was a statistically significant difference in the average score of the desire for divorce before the intervention and after the intervention, as well as one month later. However, while the average score after the intervention and one month later did not display a significant difference, the average score before the intervention and one month later exhibited statistical significance (*P* < 0.0001). Similarly, in the control group, the desire for divorce scores remained constant across the three stages of assessment (*P* > 0.05) (Table 6; Fig. 2).

**Table 6:** Comparison of the average score of the desire for divorce in the groups and over time

Desire for Divorce	Cognitive behavioral group	Schema-based group	Control group	F	*P*-value
**Before intervention**	59.85 ± 9.98	63.85 ± 13.91	57.6 ± 11.6	1.12	0.345
**One week after intervention**	36.9 ± 4.19	36.3 ± 3.68	58.1 ± 12.7	25.8	*P* < 0.0001
**One month after intervention**	35.4 ± 3.92	35.7 ± 3.75	58.86 ± 17.03	32.52	*P* < 0.0001
	*P* < 0.0001	*P* < 0.0001	*P* > 0.05	---	---

### Desire for divorce boredom score marital satisfaction scores

**Fig. 2:**
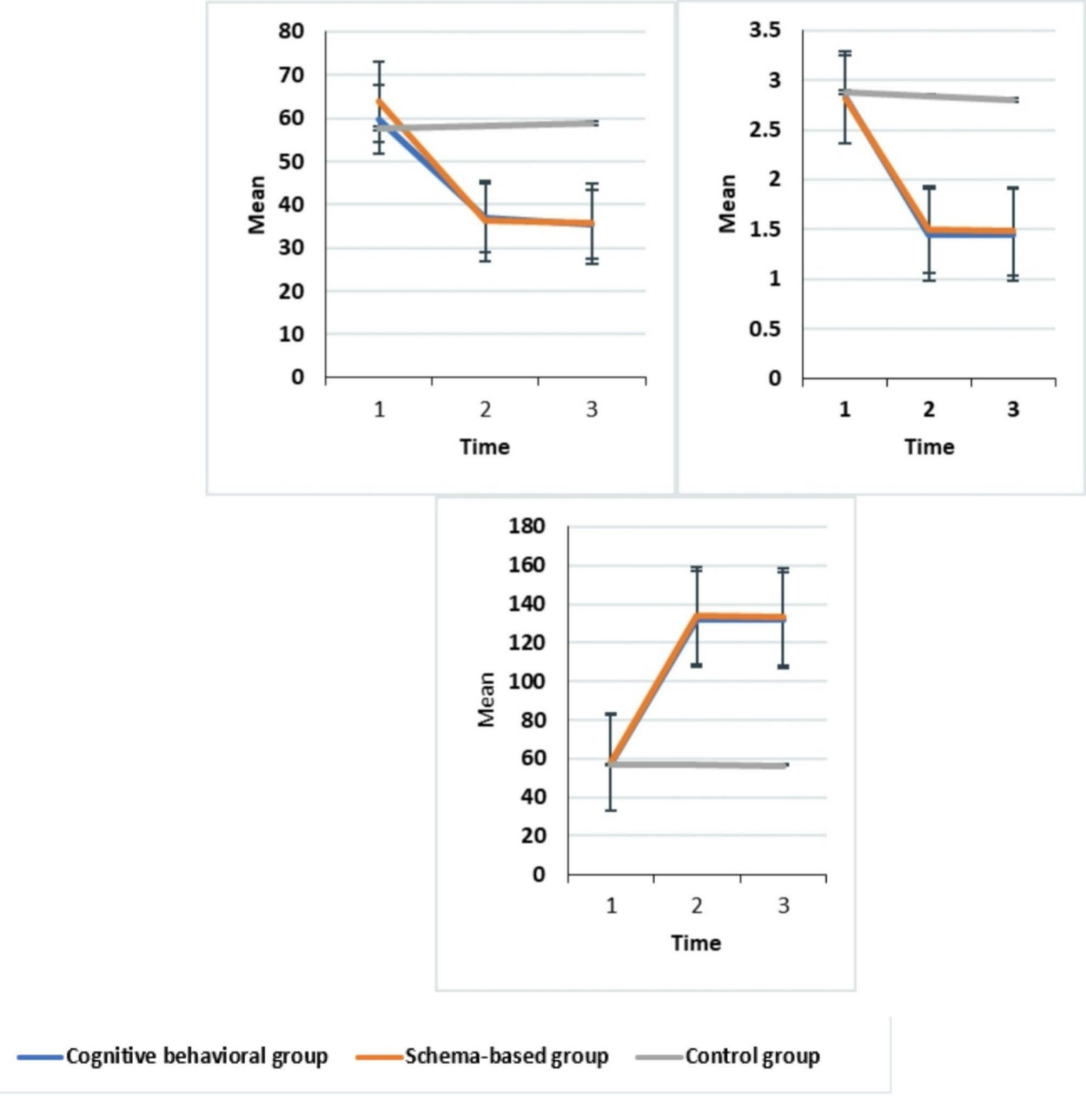
Comparison of the average score of boredom, the desire for divorce and marital satisfaction scores in the study groups over time

## Discussion

The aim of this study was to compare the effectiveness of cognitive-behavioral couple therapy and schema-based couple therapy in reducing divorce desire and marital burnout. The results of the study showed that after the training sessions, divorce desire and burnout decreased and marital satisfaction increased in the intervention groups compared to the control group.

In the context of marital boredom, couples often experience a decline in intimacy over time, leading to diminished emotional attachment and feelings of alienation, disinterest, and indifference towards each other, often accompanied by the emergence of negative emotions [17].

The findings revealed that cognitive-behavioral couple therapy led to a decrease in marital boredom and the inclination toward divorce within the experimental group, both immediately after treatment and during the follow-up period, in contrast to the control group. These findings align with previous studies by Ricardo Silva [20], Pirfalak et al. [7], Maleki et al. [9], KhanjaniVeshki et al. [21], and Nilsson et al. [22].

Cognitive-behavioral couple therapy plays a vital role in evaluating and reshaping maladaptive thoughts and behaviors to establish a healthy relationship dynamic between partners. Through cognitive techniques that involve examining thoughts, weighing the pros and cons of these thoughts, reframing negative narratives, fostering constructive dialogues, and enhancing couples’ self-awareness, the therapy aims to shift individuals’ perspectives from negative to positive. By delving into the emotion-thought-behavior cycle, cognitive-behavioral therapy addresses both personal and interpersonal challenges within couples, highlighting how cognitive distortions contribute to dysfunctional emotions, feelings, and behaviors. Ineffective communication patterns have the potential to exacerbate anger and diminish the willingness to sustain a marital bond [20].

Hence, it is imperative to address these issues and enhance communication through interventions. Training programs integrated into the cognitive aspect of therapy involve cognitive restructuring, handling negative emotions, problem-solving techniques, adopting effective coping mechanisms, positive visualization, and refining both verbal and non-verbal communication skills. Promoting active listening also plays a crucial role in alleviating negative thoughts and behaviors, thereby fostering a more positive relational atmosphere. Therefore, in this study, cognitive-behavioral couple therapy effectively reduced marital boredom and diminished the desire for divorce. These findings indicate that this approach significantly enhances the relationships of couples [23].

In childhood, humans have five fundamental needs, including secure attachment, self-dependence (independence), freedom to express needs, spontaneity and pleasure and self-control. The degree to which these needs are fulfilled varies from person to person, with psychologically healthy individuals consistently meeting these emotional requirements. Neglecting or diminishing these needs can result in the formation of schemas, which are later activated in adulthood, evoking emotional memories from childhood. Essentially, schemas symbolize the unresolved childhood issues that continue to affect individuals into adulthood [24].

Schema therapy plays a significant role in marital relationships. Yang’s schema therapy targets cognitive, emotional, and behavioral domains. This research’s findings suggest that individuals contemplating divorce may exhibit a tendency to withhold positive emotions even in response to positive behavior from their partner. Instead, they often react swiftly and negatively to any perceived negative behavior from their spouse, sometimes attempting to counterbalance these negative interactions. This negative reactivity can escalate into hostile behaviors, leading to difficulties in managing negative emotions and fostering a pattern of emotional distancing over time. In couple’s therapy employing schema therapy, couples explore the roots of such negative behaviors, acknowledging the evolutionary origins of each schema. By comprehending that current behaviors are rooted in past thoughts stemming from childhood traumas, couples can address these issues through education and schema therapy techniques. Consequently, they can identify and implement healthier alternatives for these schemas.

Schema therapy techniques empower couples to prevent the activation of maladaptive schemas and cultivate appropriate behaviors and thoughts. These techniques, such as writing a letter to an affected person, empty chair exercise, engaging in mental visualization, and creating training cards, aid in reframing negative patterns and promoting healthier relational dynamics. Consequently, schema-based couple therapy has the potential to alleviate marital boredom and reduce the desire for divorce by addressing marital challenges, and enhancing relationships.

The research findings indicate that schema therapy for couples effectively minimizes marital boredom and diminishes the desire for divorce, aligning with studies conducted by Panahifar et al. [24], Fani Sobhani et al. [16], Mahnai et al. [25], Valizadeh et al. [10], Momeni et al. [26], Hemmati et al. [10], Cheshmeh Nooshi et al. [8], Ahmadzadehaghdah et al. [9] and Moradi Vafa et al. [27]. The schema therapy approach integrates cognitive, behavioral, experiential, and relational techniques to challenge maladaptive schemas underlying negative thoughts. By addressing and processing these schemas, particularly those originating from childhood experiences that hinder emotional expression and warmth, couples can rekindle their desire to stay together, thereby reducing the likelihood of divorce and fostering psychological well-being.

This research, like other researches, had limitations. The therapy sessions were conducted with the presence of both couples and it seemed that some of them considered the presence of their spouses as an obstacle to their freedom of expression. Due to the fact that in this research, the data collection tool was a questionnaire, people may have answered the questions with a bias. Also, another limitation of this study is the reliance on voluntary and available participants from a specific geographic region, which may restrict the generalizability of the results to a broader population. It is also suggested to compare the effectiveness of CBCT and schema-based couple therapy indifferent cities of Iran and investigate the effect of these two approaches on other variables, as well. The findings of this study can be effective in couples’ conflicts reduction. The results of the present study can also be used at family consultation centers, mental hygiene services center, centers of crisis intervention, and family courts.

This study, like other studies, had some limitations. The therapy sessions were conducted with the presence of both couples, and some of them seemed to consider the presence of their spouses as an obstacle to their freedom of expression. Given that the data collection tool in this study was a questionnaire, people may have responded with bias to the questions.

## Conclusion

The study’s results reveal that both cognitive-behavioral methods and schema therapy within couple’s therapy were equally effective in tackling marital boredom and reducing the inclination towards divorce. Through a comprehensive approach integrating cognitive, emotional, and behavioral adjustments, along with interventions aimed at disrupting negative behavioral cycles, establishing boundaries through re-parenting techniques, utilizing tools such as training cards and engaging in imaginative dialogues, couples can significantly enhance their relationships. Consequently, these therapeutic modalities hold promise as valuable resources in counseling and divorce centers, offering avenues to mitigate divorce rates, resolve relationship challenges, improve communication, and bolster commitments between partners.

## Data Availability

No datasets were generated or analysed during the current study.
